# Serum S1P level in interstitial lung disease (ILD) is a potential biomarker reflecting the severity of pulmonary function

**DOI:** 10.1186/s12890-024-03081-y

**Published:** 2024-06-04

**Authors:** Fangping Ding, Zhenyang Wang, Jing Wang, Yingmin Ma, Jiawei Jin

**Affiliations:** 1 Department of Respiratory and Critical Care Medicine, Beijing Youan Hospital, Beijing Institute of Hepatology, Capital Medical University, N0. 5 Jingyuan road, Beijing, China; 2 Department of Respiratory and Critical Care Medicine, Beijing Institute of Respiratory Medicine and Beijing Chao-Yang Hospital, Capital Medical University, Beijing, China; 3 The Clinical Research Center, Beijing Chaoyang Hospital, Capital Medical University, Beijing, 100043 China

**Keywords:** Sphingosine-1-phosphate (S1P), Connective tissue disease-associated interstitial lung disease, Idiopathic pulmonary fibrosis, Pulmonary function

## Abstract

**Background:**

sphingosine-1-phosphate (S1P), a naturally occurring sphingolipid, has been involved in pulmonary interstitial remodeling signaling. However, no study has examined its clinical merits for interstitial lung disease (ILD). This study aimed to investigate the serum level of S1P in ILD patients and its clinical correlation with the severity of disease in the two main types of ILDs: the IPF and the CTD-ILD patients.

**Methods:**

This retrospective observational pilot study included 67 ILD patients and 26 healthy controls. These patients were stratified into the IPF group (35) and the CTD-ILD group (32). The severity of ILD was evaluated through pulmonary function indicators and the length of hospital stay.

**Results:**

Serum S1P level was statistically higher in ILD patients than in health control (*p* = 0.002), while the Serum S1P levels in CTD-ILD and IPF patients were comparable. Serum S1P level further showed statistically negative correlation with pulmonary function indexes (TLC% pred, FVC% pred and FEV1% pred) and positive correlation with length of hospital stay (*r* = -0.38, *p* = 0.04; *r* = -0.41, *p* = 0.02, *r* = -0.37, *p* = 0.04; *r* = 0.42, *p* = 0.02, respectively) in CTD-ILD patients, although serum S1P level was not significantly correlated with inflammatory indexes. The IPF patients failed to exhibit a significant correlation of serum S1P level with pulmonary function and length of hospital stay.

**Conclusions:**

Serum S1P level might be a clinically useful biomarker in evaluating the severity of CTD-ILD patients rather than IPF patients.

## Introduction

Interstitial lung disease (ILD) is characterized as a group of diffuse parenchymal lung disorders that impair lung compliance, leading to ventilatory depression and respiratory restriction [1, 2]. The two most common clinical subtypes of ILD patients are idiopathic pulmonary fibrosis (IPF) and connective tissue disease-associated interstitial lung disease (CTD-ILD) [1]. IPF and CTD-ILD patients (especially with rheumatoid arthritis) can exhibit similar usual interstitial pneumonia (UIP) patterns in the chest computed tomography (CT), but their pathogenetic mechanisms, therapeutic strategies, and prognosis are distinct [3–5]. ILD has a high morbidity and mortality due to heterogeneous pathogenesis, and thus earlier diagnosis and evaluation are important for improving its treatment and prognosis [2]. The severity of respiratory restriction in ILD patients, which is usually correlated with poor prognosis, is classified based on pulmonary volume measurements, including forced vital capacity (FVC), total lung capacity values (TLC), and forced expiratory volume (FEV1), and diffusing capacity (carbon monoxide diffusion, DLCO) [6, 7]. Therefore, an effective biomarker to monitor respiratory function change is essential for clinicians.

Sphingosine-1-phosphate (S1P), a normal metabolic product of cell membrane sphingolipids, regulates various cellular functions, including proliferation, apoptosis, endothelial barrier function, angiogenesis, and inflammation [8, 9]. S1P is found enriched in circulatory fluid and involved in embryonic development and postnatal diseases, including pulmonary interstitial remodeling, such as Serum S1P level is increased in IPF patients [9–12]. Bench studies indicate that S1P facilitates lung fibrosis by enhancing alveolar epithelial to mesenchymal transition (EMT, myofibroblast activation) and regulating alveolar endothelial function [10, 11, 13]. Since S1P could be an effective therapeutic target for IPF, S1P agonists have been tested in some bench research, including Fingolimod, Siponimod and Ponesimod [13, 14]. However, to date, no studies have investigated the clinical correlation between serum S1P level and ILD severity, which is critical to provide clinical evidence for evaluating the merits of S1P in ILD.

Herein, we performed a retrospective observational pilot study on the existing ILD patient serum samples to compare the serum S1P levels in ILD patients and healthy subjects. Given the distinct pathologists between IPF and CTD-ILD, we hypothesize that serum S1P level may show different diagnostic value in these two groups. To test this hypothesis, we compared the serum S1P level in these two types of ILD patients and further examined the statistical correlations of S1P level to severity in these two types of ILD patients. Our findings could shed light on developing earlier biomarkers for the progression of ILD.

## Methods

### Study design

This was a pilot retrospective study on existing ILD data, in which serum samples and clinical data were collected between January 2019 to December 2021 in the Department of Respiratory and Critical Care Medicine, Beijing Chao-Yang Hospital. This study was approved by the Ethics Committee of Beijing Chao-Yang Hospital (2021-KE-295) and carried out in accordance with relevant guidelines and regulations, and written informed consent was obtained from all subjects. A total of 196 patients with ILD presenting UIP or probable UIP patterns in chest CT examination were enrolled, and patients with acute infection, cancer, corticosteroids using history, Sarcoidosis, pneumoconiosis, or incomplete data were excluded. The 67 ILD patients were included in the analyses. IPF and CTD-ILD patients were identified based on the published criteria of the corresponding societies [14–20]. The healthy subjects were volunteers with a normal chest CT examination from the physical examination center during the same period.

### Data collection

The patients with the initial diagnosis of CTD-ILD or IPF were enrolled and their serum samples were collected at admission; the pulmonary function data were collected within two days after admission. The clinical data included gender, age, smoking history, vital signs, underlying diseases, laboratory examining data and pulmonary function test (PFT) were obtained from medical records. Serum S1P (ng/mL) was measured through Elisa kits from Cloud-clone.

### Statistical analysis

The continuous variables in normal distribution were presented as means ± standard deviations (SD), and the two-tailed student’s t-test was used to compare the two groups. The standard difference in serum S1P level between the two groups was expressed by effect size (Cohen’s d). The continuous variables in non-normal distribution were presented as the median and interquartile range (IQR, 25th-75th percentiles), and the Mann-Whitney U test was performed to compare the two groups. The statistical comparison of three groups was performed using one-way ANOVA followed by post hoc multiple comparison tests where appropriate. Categorical variables were depicted as numbers and percentages and analyzed with chi-squared or Fisher’s exact tests. Spearman correlation analysis was used to evaluate the correlation between serum S1P and the length of hospital stay, and other laboratory indexes. After the LOG10 transformation of serum S1P level (ng/ml), the simple linear regression was further used to determine the significant association of serum S1P levels to the length of hospital stay and the pulmonary function indexes. Residual analysis was used for assessing the assumptions of linear regression. Multiple linear regressions were also performed to examine the independent associations of S1P and pulmonary function indexes according to CRP and Platelet in the IPF and CTD-ILD groups, respectively. A two-sided p-value of < 0.05 was considered statistically significant, and all analyses were performed with SPSS (version 19.0, SPSS, Chicago, IL, USA).

## Results

### The serum S1P level was significantly increased in CTD-ILD and IPF patients

**Fig. 1:**
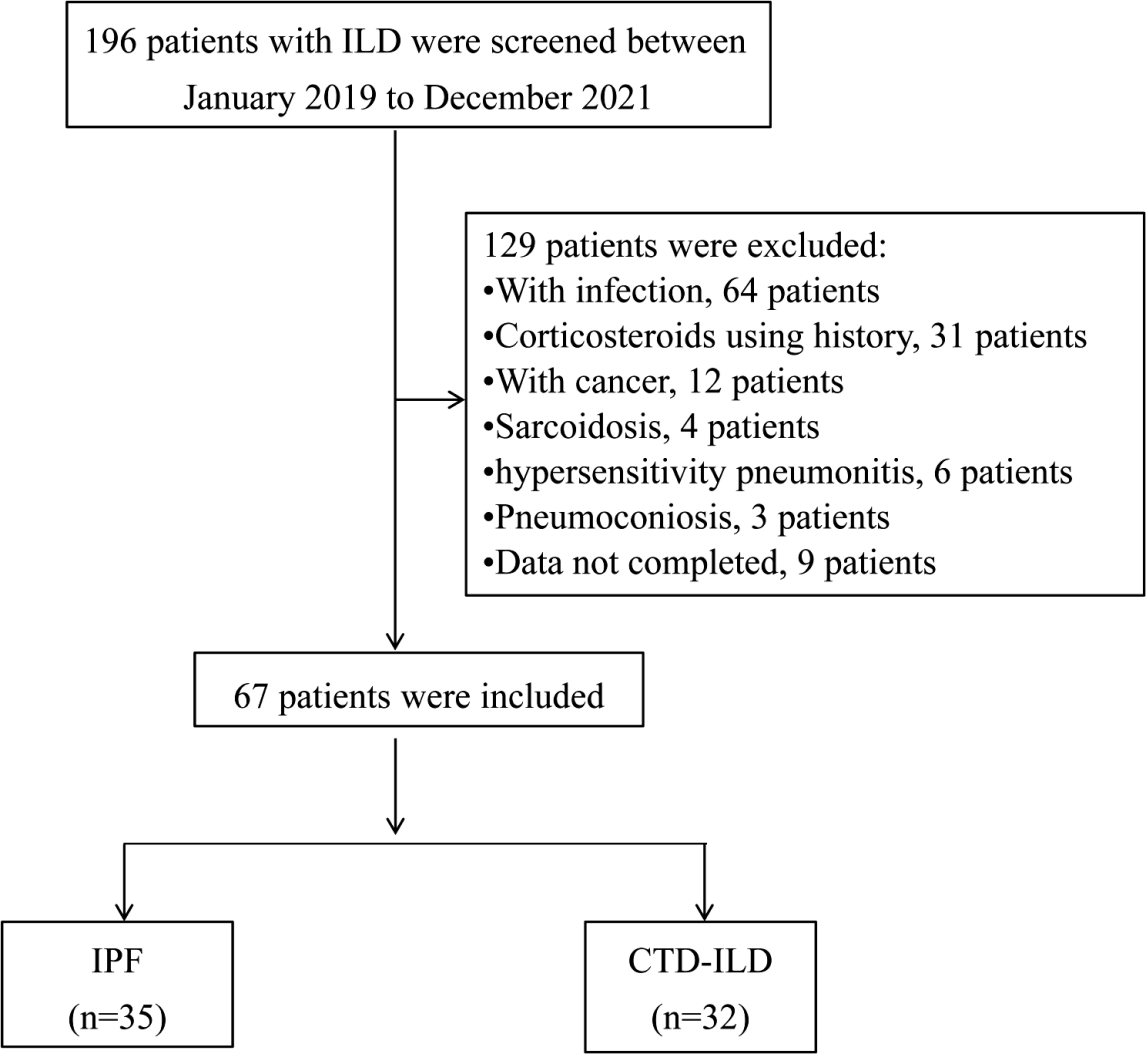
Flowchart of patient enrollment. ILD, interstitial lung disease; IPF, idiopathic pulmonary fibrosis; CTD-ILD, connective tissue disease-associated interstitial lung disease

**Fig. 2:**
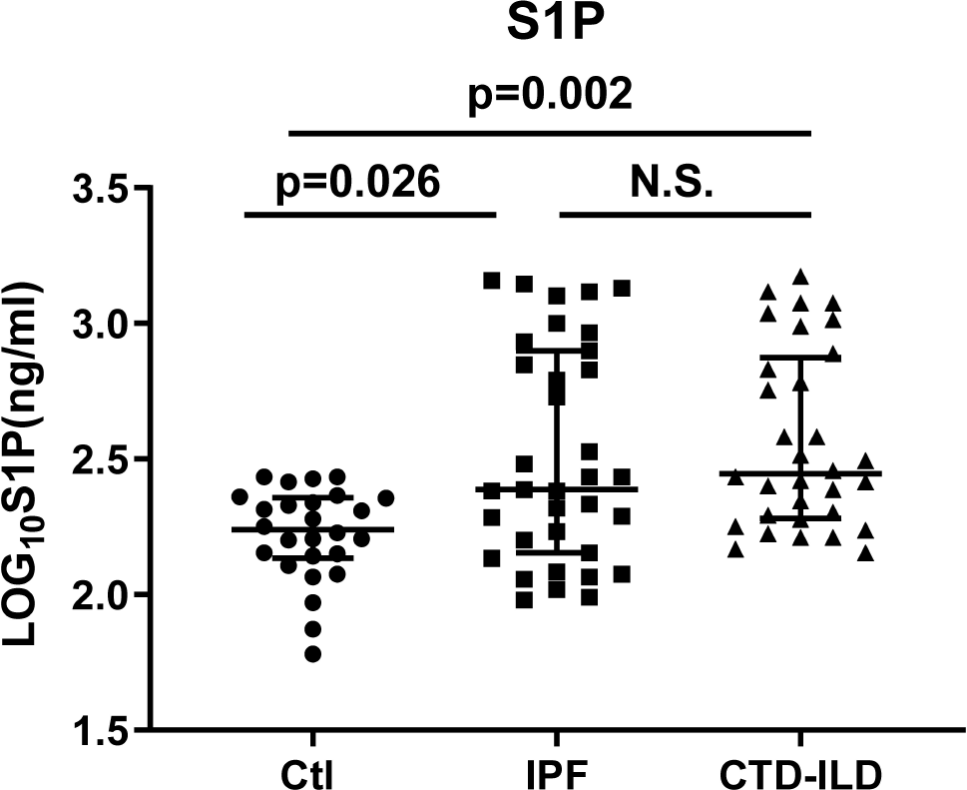
Serum S1P level in healthy controls, ILD patients with IPF or CTD-ILD. Ctl, healthy controls; IPF, idiopathic pulmonary fibrosis; CTD-ILD, connective tissue disease-associated interstitial lung disease; ns, no significance

This pilot retrospective study enrolled 26 healthy subjects and 67 ILD patients, including 35 IPF and 32 CTD-ILD patients (Fig. 1, a flowchart of patient enrollment). The baseline clinical data of healthy people and ILD patients at admission are shown in Tables 1 and 2. There was no significant difference in sex, age, white blood cell, neutrophil granulocyte, lymphocyte, hemoglobin and platelet between health and ILD patients, whereas the serum S1P level was significantly increased in ILD patients (*P* = 0.002) and Cohen’s d = 0.75 for the difference of serum S1P between ILD patients and healthy controls. In addition, IPF and CTD-ILD groups exhibited comparable levels of serum S1P, though S1P was significantly increased in both groups (*P* = 0.026 and *P* = 0.002, respectively). Of note, the statistic P value versus controls was relatively lower in CTD-ILD patients than in the IPF group (Fig. 2).

**Table 1:** Comparison of serum S1P level between healthy controls or ILD patients

Variables	Healthy controls (*n* = 26)	ILD patients (*n* = 67)	*P*
Male, n (%)	11 (42.3)	36 (56.3)	0.550
Age (years)	62 ± 9	64 ± 10	0.120
Current smoker, n (%)	N/A	12 (18.8)	
Smoking index	N/A	30 (0 ~ 487)	
*White blood cell (×10* ^*9*^ */L)*	*6.17 ± 1.03*	*6.97 ± 2.10*	*0.020*
Neutrophil granulocyte (×10^9^/L)	3.43 (2.82 ~ 3.98)	3.84 (2.98 ~ 4.79)	0.097
Lymphocyte (×10^9^/L)	2.02 ± 0.40	1.88 ± 0.67	0.209
Haemoglobin (g/L)	133.96 ± 11.36	133.48 ± 17.01	0.896
Platelet (×10^9^/L)	234.3 ± 42.7	212.9 ± 53.4	0.168
*S1P level in serum (ng/ml)*	*178.3 (136.1 ~ 227.5)*	*252.7 (162.3 ~ 697.3)*	*0.002*

ILD, interstitial lung disease; N/A, not applicable

**Table 2:** Baseline characteristics of the ILD patients

Variables	Overall (*n* = 67)	IPF (*n* = 35)	CTD-ILD (*n* = 32)	*P* (IPF versus CTD-ILD)
*Male, n (%)*	*36 (56.3)*	*24 (68.6)*	*12 (37.5)*	*0.029*
Age (years)	64 ± 10	66 ± 8	62 ± 11	0.418
Current smoker, n (%)	12 (18.8)	7 (20.0)	5 (17.2)	0.78
Smoking index	30 (0 ~ 487)	100 (0 ~ 500)	0 (0 ~ 475)	0.465
Underlying diseases, n (%)				
Hypertension	22 (34.4)	16 (45.7)	6 (20.7)	0.053
Diabetes	19 (29.7)	12 (34.3)	7 (24.1)	0.376
Cardiovascular disease	18 (28.1)	11 (31.4)	7 (24.1)	0.518
Cerebrovascular disease	6 (9.4)	4 (11.4)	2 (6.9)	0.536
Subtypes of CTD, n (%)				
Rheumatoid arthritis		N/A	10 (31.3)	
Sjogren’s syndrome		N/A	9 (28.1)	
Systemic lupus erythematosus		N/A	5 (15.6)	
Dermatomyositis		N/A	2 (6.3)	
Systemic Sclerosis		N/A	2 (6.3)	
Mixed connective tissue disease		N/A	4 (12.5)	
Radiologic patterns of ILD, n(%)				0.08
UIP		33 (94.3)	23 (79.3)	
Probable UIP		2 (5.7)	6 (20.7)	
The onset of symptoms to hospital admission (days)	60 (20 ~ 173)	60 (30 ~ 123)	65 (19 ~ 372)	0.632
Length of hospital stay	13 (8 ~ 10)	9 (7 ~ 14)	13 (9 ~ 11)	0.524
Laboratory data at admission				
Lactate (mmol/L)	1.00 (0.80 ~ 1.40)	1.20 (0.90 ~ 1.50)	0.90 (0.75 ~ 1.20)	0.107
White blood cell(×10^9^/L)	6.97 ± 2.10	6.89 ± 1.82	7.0 ± 2.42	0.080
Neutrophil granulocyte (×10^9^/L)	3.84 (2.99 ~ 4.79)	3.87 (3.23 ~ 4.76)	3.79 (2.97 ~ 5.18)	0.599
Lymphocyte (×10^9^/L)	1.88 ± 0.67	1.92 ± 0.67	1.82 ± 0.68	0.951
Haemoglobin (g/L)	133.18 ± 17.02	136.02 ± 15.41	130.5 ± 18.6	0.184
Platelet (×10^9^/L)	217.92 ± 53.38	208.82 ± 43.70	228.90 ± 62.17	0.144
C-reactive protein (mg/L)	5.0 (5.0 ~ 10.8)	5.0 (1.0 ~ 9.0)	6.0 (5.0 ~ 13.8)	0.071
Procalcitonin (ng/ml)	0.05 (0.05 ~ 0.05)	0.05 (0.05 ~ 0.05)	0.05 (0.05 ~ 0.05)	0.876
*ESR (mm/h)*	*20.0 (9.3 ~ 40.0)*	*15.0 (5.0 ~ 35.0)*	*30.0 (10.0 ~ 47.5)*	*0.036*
ALT (U/L)	16.5 (11.7 ~ 21.0)	17.6 (12.0 ~ 23.6)	16.0 (10.8 ~ 20.0)	0.128
AST (U/L)	20.1 (17.1 ~ 24.4)	20.1 (17.0 ~ 25.5)	20.0 (17.7 ~ 24.3)	0.898
Creatinine (umol/L)	69.8 ± 13.7	72.3 ± 14.2	66.7 ± 12.6	0.529
D-Dimer (mg/L)	0.15 (0.08 ~ 0.26)	0.13 (0.07 ~ 0.25)	0.16 (0.095 ~ 0.40)	0.292
Pulmonary function test				
TLC% pred	73.8 ± 12.4	73.7 ± 11.4	73.8 ± 13.8	0.282
FVC% pred	82.9 ± 17.9	85.6 ± 16.2	79.6 ± 19.53	0.590
FEV1% pred	85.4 ± 16.1	88.4 ± 15.3	81.8 ± 16.5	0.793
DLCO% pred	66.1 ± 11.6	68.5 ± 11.6	63.1 ± 11.1	0.874
S1P level in serum (ng/ml)	252.7 (162.3 ~ 697.3)	244.49 (142.8 ~ 793.2)	264.0 (175.6 ~ 587.0)	0.931

IPF, idiopathic pulmonary fibrosis; CTD-ILD, connective tissue disease-associated interstitial lung disease; UIP, usual interstitial pneumonia; ESR, erythrocyte sedimentation rate; AST, aspartate transaminase; ALT, alanine aminotransferase; TLC% pred, predicted total lung capacity; FVC% pred, predicted forced vital capacity; DLCO% pred, predicted lung diffusing capacity for carbon monoxide. * Student t-test, # Mann-Whitney test

### The correlations of serum S1P level with pulmonary function and inflammatory indexes in CTD-ILD and IPF patients

To clarify whether the increased S1P in circulation would be correlated with clinical conditions or the severity of ILD, we examine the correlations of S1P with the length of hospital stay, pulmonary function indexes and inflammation-associated indicators in IPF and CTD-ILD groups, respectively. The heterogeneous CTD characteristics of CTD-ILD patients were shown in Table 2. There was no statistically significant difference in most clinical and laboratory indicators between CTD-ILD and IPF patients, except gender (*P* = 0.029) and ESR (*P* = 0.036) (Table 2).

Interestingly, in CTD-ILD patients, Spearman correlation analysis identified negative correlations of serum S1P level with pulmonary function indexes (TLC% pred, FVC% pred and FEV1% pred) and Platelet level and positive correlation with length of hospital stay, although serum S1P level was not significantly correlated with inflammatory indexes including CRP, ESR and hemoglobin serum levels (Table 3). Conversely, in IPF patients, there was no significant correlation between serum S1P level with pulmonary function and the length of hospital stay, though a negative correlation with CRP and positive correlation with AST (*r* = -0.62, *p* < 0.01; *r* = 53, *p* = 0.024, respectively) (Table 3).

**Fig. 3:**
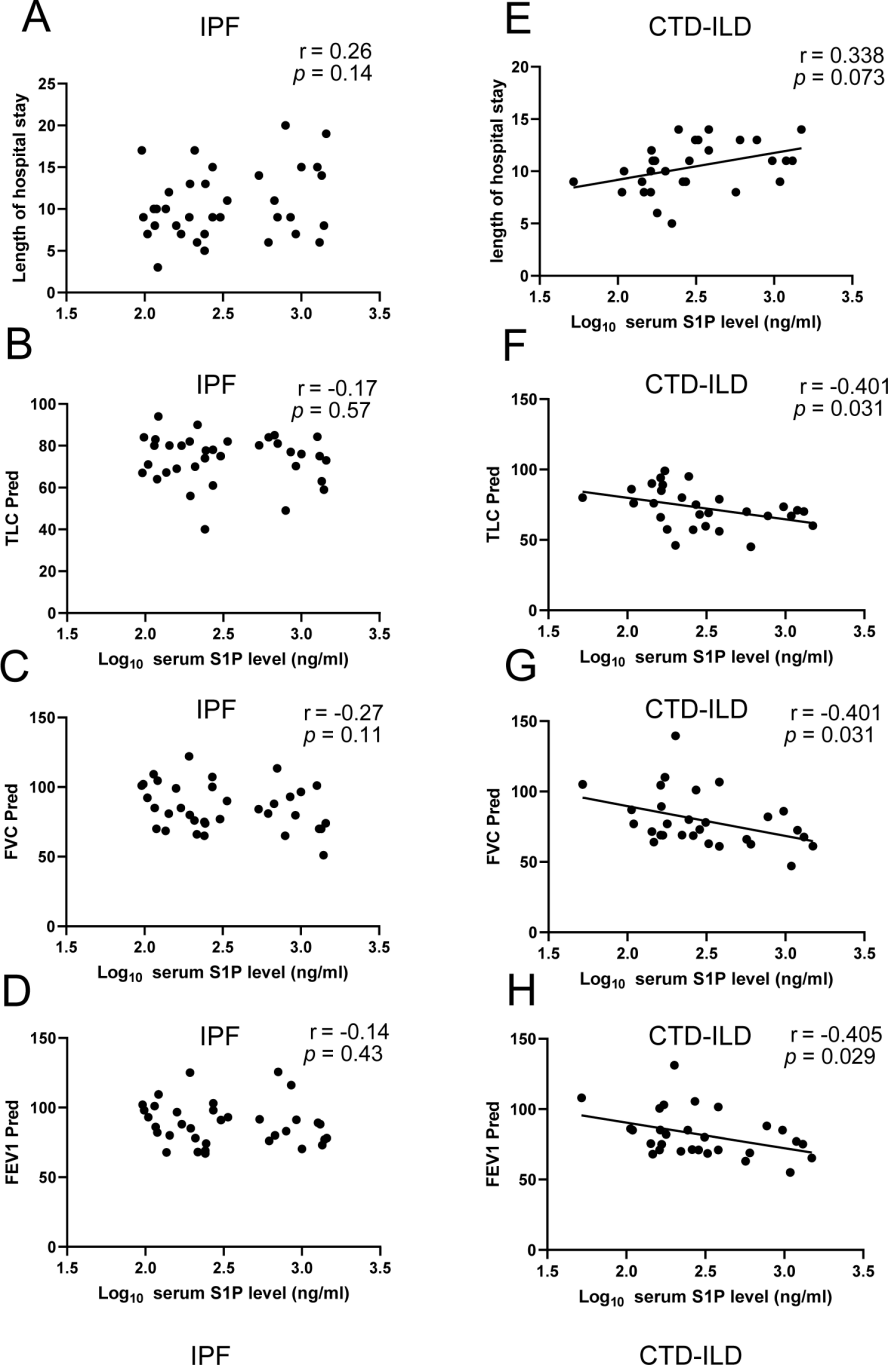
Scatter diagram for the relationship between serum S1P level and pulmonary function indexes by linear regression analyses (TLC% pred, FVC% pred and FEV1% pred) in IPF patients (**A**, **B**, **C** and **D**) and CTD-ILD patients (**D**, **E**, **F** and **G**). IPF, idiopathic pulmonary fibrosis; CTD-ILD, connective tissue disease-associated interstitial lung disease; TLC% pred, predicted total lung capacity; FVC% pred, predicted forced vital capacity; FEV1%pred, Forced expiratory volume in the first second

To further study the value of serum S1P level for predicting the severity of CTD-ILD, we conducted linear regression to the analysis of the linear correlation of serum SIP to the length of hospital stay and (TLC% pred, FVC% pred and FEV1% pred) (Fig. 3). We further conducted the adjusted correlation analysis by multiple linear regression according to parameters (Platelet), which was statically correlated with S1P to the severity of the CTD-ILD. The LOG10 transformation of serum S1P level is a way to change the scale of a variable to make it more amenable to linear regression. The linear regression results showed that LOG10 serum S1P was statically correlated with pulmonary function indexes TLC% pred (β=-0.401; 95% confidence interval=-28.8, -1.491; *p* = 0.03), FVC% pred (β= -0.40; 95% confidence interval=-40.39, -2.087; *p* = 0.031), FEV1% pred (β= -0.405; 95% confidence interval=-34.23, -1.98; *p* = 0.029), but it failed to correlated with length of hospital stay (β = 0.338; 95% confidence interval=-0.231, 4.828; *p* = 0.073) in linear regression model. After adjustment for confounding factors (Platelet), serum S1P level in CTD-ILD patients consistently showed negative correlations with pulmonary function indexes TLC% pred (β=-0.427; 95% confidence interval=-30.931, -1.297; *p* = 0.034), FVC% pred (β=-0.492; 95% confidence interval=--46.126, -5.985; *p* = 0.013), FEV1% pred (β=-0.494; 95% confidence interval=-39.003, -5.141; *p* = 0.013). These interesting findings suggest the potential clinical merit of serum S1P for evaluating the pulmonary function changes and the severity of CTD-ILD patients rather than IPF patients.

**Table 3:** the correlations between serum S1P level with variables

Correlation SIP serum level (ng/ml)				
	IPF		CTD-ILD	
Variables	r	*P*	r	*P*
Length of hospital stay	0.184	0.29	*0.42*	*0.02*
Pulmonary function test				
TLC% pred	-0.09	0.60	*-0.38*	*0.04*
FVC% pred	-0.29	0.08	*-0.41*	*0.02*
FEV1% pred	-0.22	-0.20	*-0.37*	*0.04*
DLCO% pred	0.03	0.85	-0.02	0.91
DLCO pred/ FVC pred			0.453	
Lactate (mmol/L)	-0.07	0.68	0.01	0.96
White blood cell	0.07	0.68	0.0892	0.65
neutrophil-to-lymphocyte ratio (NLR)	0.05	0.79	-0.077	0.69
Haemoglobin	0.22	0.20	0.02181	0.91
Platelet	-0.05	0.78	*-0.4536*	*0.01*
*C-reactive protein*	*-0.62*	*< 0.0001*	-0.3342	0.08
ESR (mm/h)	-0.04	0.848	-0.2904	0.13
ALT (U/L)	0.18	0.31	-0.09081	0.64
*AST (U/L)*	*0.35*	*0.04*	-0.08527	0.66
Creatinine (umol/L)	-0.25	0.15	-0.1746	0.37
D-Dimer (mg/L)	0.07	0.69	-0.236	0.22

## Discussion

This present study, for the first time, investigated the clinical value of serum S1P in stratified ILD patients: IPF and CTD-ILD groups (patients selected had a UIP or probable UIP pattern on CT examination, CTD-UIP). The primary findings are as follows: the serum S1P level was significantly increased in both IPF and CTD-UIP patients, while IPF and CTD-UIP showed comparable S1P levels; In CTD-UIP patients, but not IPF patients, serum S1P level showed significantly negative correlation with pulmonary function change and positive correlation the length of hospital stay, which represents the severity of CTD-UIP; the association value of serum S1P with pulmonary function was further confirmed by linear regression.

The severity of respiratory restriction in ILD patients is usually correlated with poor prognosis, and thus an effective biomarker to monitor respiratory function change is essential to improve the clinical outcome. while several plasma biomarkers, such as CXCL13, CA-125, MMP7, SP-D, YKL-40, and VCAM-1, have been associated with prognostic survival in patients with ILD, these biomarkers are rarely utilized for assessing pulmonary function in clinical practice [21]. Pulmonary function assessment for ILD patients is typically based on lung volume measurements (FVC, TLC and FEV1) and diffusing capacity (DLCO) [6, 7]. In this study, we indicated a significant negative correlation between increased serum S1P and changes in lung volume indexes in CTD-UIP patients. This functional association is consistent with the conclusion in previous bench studies that S1P as a pro-fibrotic factor promoted lung interstitial remodeling [10–12]. Our pilot study advanced the clinical importance of circulating S1P during chronic pulmonary remodeling. Notably, serum S1P failed to correlate with alveolar diffusing capacity, suggesting that S1P is sensitive to remodeling-associated lung volume change rather than alveolar diffusing. This was supported by the observation that SIP is correlated with PFT indexes in CTD-UIP patients, but not in IPF patients. Chronical IPF patients primarily present diffusing dysfunction resulting from fibrotic remodeling including alveolar septal thickness, fibrotic foci formation, and fibrotic plaque around the edge of the lung lobe. In contrast, both immune-inflammatory deregulation-induced pulmonary interstitial remodeling and fibrogenesis are pathogenic mechanisms of CTD-UIP. The distinct pathogenetic mechanisms could explain different clinical value of serum S1P in these two types of ILD patients.

Previous studies highlighted some circulating plasma biomarkers for ILD prognosis, such as CXCL13, CA-125, MMP7, SP-D, YKL-40 and VCAM-1, all of which are proteins associated with inflammation and interstitial remolding [21]. Our pilot study suggests the clinical value of serum S1P, a lipid signal molecule involved in various cellular functions and diseases, including fibrosis and inflammation [8, 9]. This novel lipid biomarker showed a clinical relationship with pulmonary functional depression. It was also consistently correlated with the length of hospital stay, suggesting S1P could be a useful biomarker to monitor the progression of CTD-UIP. The clinical value of S1P is consistent with previous bench studies, in which S1P plays a profibrotic role in lung fibrosis through enhancing TGF-β1-induced EMT, while the S1P inhibition by S1P lyase overexpression attenuates lung fibrosis [10, 11]. Conversely, S1P has also been shown to be an antagonist of IPF by strengthening the integrity of the endothelial barrier via S1P receptor 1 (S1PR1)/Rac pathways and constricting pulmonary inflammation [13]. This contradiction suggests a complicated role of S1P in IPF pathogenesis, which could partially explain the non-correlation of serum S1P with pulmonary function in IPF patients.

Taken together, our results showed that increased serum S1P was correlated with pulmonary functional depression CTD-UIP patients rather than IPF patients, and it might be a clinically useful biomarker in evaluating the severity of CTD-UIP patients due to the involvements of S1P both in immune-inflammatory response and fibrogenesis, both of which are the primary pathogenic mechanism of CTD-UIP. However, a prospective study enrolling more subjects is needed to validate the clinical value of S1P.

### Limitations

There were some limitations of our present pilot study: (1) This was a retrospective study that included data from a single-center cohort and enrolled a small number of patients. (2) The limited sample size does not allow for further stratified analysis examining whether the serum Rcn3 levels are different according to the types of CTD, such as rheumatoid arthritis, systemic sclerosis, and inflammatory myositis. (3) We missed a control group with CTD without ILD because most patients did not have pulmonary function test data during this retrospective study.

## Conclusions

Serum S1P level was significantly increased in both IPF and ILD patients. In CTD-UIP patients, but not IPF patients, serum S1P level showed a significantly negative correlation with pulmonary function change and a positive correlation with the length of hospital stay, representing the severity of CTD-UIP. Serum S1P level might be a clinically useful biomarker in evaluating CTD-UIP rather than IPF patients.

## Data Availability

The data collected and analyzed during the current study are available from the corresponding author on reasonable request.

## Abbreviations

ILD: Interstitial lung disease
UIP: Usual interstitial pneumonia
CTD-ILD: Connective tissue disease-associated interstitial lung disease
CTD-UIP: Connective tissue disease-associated interstitial lung disease with UIP or probable UIP pattern on CT examination
S1P: Sphingosine-1-phosphate
S1PR1: Sphingosine-1-phosphate receptor 1
TLC% pred: Predicted total lung capacity
FVC% pred: Predicted forced vital capacity
DLCO% pred: Predicted lung diffusing capacity for carbon monoxide
CRP: C-reactive protein
PCT: Procalcitonin
ALT: Alanine transaminase
AST: Aspartate aminotransferase
ESR: Erythrocyte sedimentation rate
NLR: Neutrophil-to-lymphocyte ratio
